# Explaining seasonality increases perceived effectiveness of influenza vaccination: An experimental study

**DOI:** 10.1111/bjhp.12770

**Published:** 2024-11-28

**Authors:** Lisa Felgendreff, Felix G. Rebitschek, Parichehr Shamsrizi, Mattis Geiger, Mirjam A. Jenny, Cornelia Betsch

**Affiliations:** ^1^ Institute for Planetary Health Behaviour, University of Erfurt Erfurt Germany; ^2^ Health Communication BNITM Bernhard Nocht Institute for Tropical Medicine Hamburg Germany; ^3^ Department of Journalism and Communication Research Hanover University of Music, Drama and Media Hanover Germany; ^4^ Harding Center for Risk Literacy, Faculty of Health Sciences Brandenburg University of Potsdam Potsdam Germany; ^5^ Max Planck Institute for Human Development Berlin Germany

**Keywords:** analogy, educational intervention, fact box, informed decision‐making, narrative, storytelling, vaccine hesitancy

## Abstract

**Background:**

Doubts regarding vaccine effectiveness may prompt people to decide against a seasonal influenza vaccination. While fact boxes show the effectiveness in terms of cases prevented, people often lack knowledge about important contextual factors, for example, why the vaccine formulation needs to be updated annually, the vaccine mechanism and relevance of the antigen–virus match. Adding such contextual information could improve effectiveness perceptions.

**Objective:**

In a preregistered online experiment, we tested whether explaining the seasonality's relevance and mechanisms behind influenza vaccine effectiveness affects people's perceptions of influenza vaccination. We compared two means of explanation (an additional expository text vs. a narrative offering an analogy to improve understanding of vaccine effectiveness) with a control condition simply providing effectiveness information.

**Design:**

Unvaccinated participants (*N* = 1554) were assigned to one of three conditions: (1) fact box only (providing the influenza vaccine's benefit–risk profile; control group), (2) fact box plus informational expository text or (3) fact box plus narrative analogy.

**Methods:**

After the experimental manipulations, participants rated the vaccine's effectiveness in preventing influenza disease and answered knowledge questions. Effects on perceived risk of vaccination and intention to get vaccinated were also explored.

**Results:**

Reading the expository text increased the perceived vaccine effectiveness and overall knowledge, while reading the narrative analogy only increased the perceived vaccine effectiveness compared with the control condition. All other dependent variables were similar in both text conditions.

**Conclusions:**

Extended explanations of vaccine effectiveness can increase perceived vaccine effectiveness. The text format chosen can affect outcomes, such as vaccine‐related perceptions or knowledge.

**Statement of Contribution**

**What is already known on this subject?**

- Influenza vaccination rates are low in relevant target groups despite official recommendations.
- The effectiveness of influenza vaccines varies seasonally.
- Lower perceived influenza vaccine effectiveness is related to a lower willingness to get vaccinated.

**What does this study add?**

- Explaining reasons for varying vaccine effectiveness increases perceived vaccine effectiveness.
- An expository text and a narrative analogy similarly increased perceived vaccine effectiveness.
- The text format influenced the performance on a knowledge test.

## INTRODUCTION

Seasonal influenza globally burdens individuals at risk, health systems, and national economies, even though vaccination offers an effective preventive measure (Iuliano et al., 2018; Macias et al., 2021; Peasah et al., 2013; Troeger et al., 2019). Many federal health authorities recommend immunization of vulnerable groups (Council of the European Union, 2009; Morales et al., 2021), yet substantial gaps in the target groups' seasonal vaccination coverage exist (e.g. Gravagna et al., 2022; Neufeind et al., 2021; Sheikh et al., 2018). Besides lack of awareness about vaccination recommendations and lingering misconceptions about the influenza vaccination, many people omit this vaccination because they perceive the vaccine's effectiveness in preventing influenza diseases to be low (Abu‐rish et al., 2016; Schmid et al., 2017). In a preregistered study, we therefore tested whether explaining the seasonality's relevance and consequences for vaccine formulation and effectiveness in an expository text (i.e. a purely informational text) or a narrative analogy can increase the perceived vaccine effectiveness and general knowledge about influenza vaccination.

Several theories about health behaviour postulate that when deciding about adopting a behaviour, the health threat and the potential countermeasure are evaluated and weighed against each other (e.g. Protection Motivation Theory, Maddux & Rogers, 1983; Health Belief Model, Rosenstock, 1974). In the case of influenza vaccination, the probability and severity of contracting influenza are weighed against the vaccine's effectiveness in preventing (severe) influenza disease and its risks (e.g. adverse events) or practical barriers to receiving a vaccination (e.g. opportunities, costs). According to these health theories, perceived vaccine benefits (such as effectiveness beliefs) predict intentions and health behaviours (e.g. vaccine uptake). However, people may hold false beliefs about vaccine effectiveness, that is, the fact, that vaccination can effectively prevent influenza (Abu‐rish et al., 2016; Schmid et al., 2017).

Interventions providing the best available evidence can decrease knowledge gaps and lead people to update their effectiveness beliefs (Cameron et al., 2013; Felgendreff et al., 2023; Mitchell et al., 2021; Mostafapour et al., 2019). One such evidence‐based way to provide effectiveness information is to contrast the probability of contracting influenza and its health outcomes in vaccinated and unvaccinated people using ‘fact boxes’ (Lühnen et al., 2015). A fact box provides the numbers for both groups in a tabular or graphical format to facilitate comparison (McDowell et al., 2016). It is comprehensible, can foster an informed decision, and increase vaccine confidence (Brick et al., 2020; McDowell et al., 2019; Rebitschek et al., 2022).

There are reasons why merely providing information on vaccine effectiveness without giving relevant context does not seem adequate. Influenza vaccines are, on average, less effective than other well‐established vaccines—a Cochrane review from 2018 reported an average of 53% prevented laboratory‐confirmed diagnoses of influenza for healthy adults (Demicheli et al., 2018)—because many influenza strains exist and their regional distribution varies seasonally. This is also why seasonal influenza vaccines must be updated annually based on the most likely circulating influenza strains of the upcoming season. Thus, vaccine effectiveness in a given season depends on how well the vaccine formulation matches the circulating influenza strains (World Health Organization [WHO], 2022). While mass media covers the (low) effectiveness and adverse events of seasonal and pandemic influenza vaccines, they rarely explain why influenza vaccines need to be updated and what causes the variability in vaccine effectiveness (Meyer et al., 2016; Murdoch & Caulfield, 2018). Therefore, the public's beliefs about influenza vaccines being an ineffective prevention measure may rest on a lack of knowledge: on why the seasonal influenza vaccine needs to be updated regularly, how it protects against an influenza disease, and that it can help even if the antigen–virus match is imperfect. Information about these aspects may enhance the perceived vaccine effectiveness and vaccine knowledge. The goal of this study is to test this assumption.

This information could be provided in different styles. For example, one could offer scientific information in explanatory texts or provide a vivid narrative to convey the same information. Reyna (2021) suggested that narratives can deliver the essential bottom‐line of scientific explanations (e.g. that vaccine effectiveness depends on the influenza strain‐vaccine formulation match). Hinyard and Kreuter (2007) defined the term *narrative* as ‘any cohesive and coherent story with an identifiable beginning, middle, and end that provides information about scene, characters, and conflict; raises unanswered questions or unresolved conflict; and provides resolution’ (p. 778). Narratives offer a familiar structure and context that makes them more comprehensible and memorable for laypersons than expository texts, whose primary function is to inform readers (Mar et al., 2021). In addition, they can stir situational interest and stimulate engagement with the topic in a nonexpert audience (Dahlstrom, 2014). Thus, narratives can be used to evoke interest in a topic or transfer factual knowledge (Lühnen et al., 2017).

Given these potential advantages, there have been attempts to convey scientific information in narrative texts (Flemming et al., 2018; Golke et al., 2019). While some studies showed similar learning outcomes (Ecker et al., 2020; Flemming et al., 2018; Mensink et al., 2021; Wolfe & Mienko, 2007; Zebregs et al., 2015), others suggest that expository texts may be more effective than narratives, for example, in stimulating the integration of new factual content with prior knowledge (Wolfe & Woodwyk, 2010). Yet, in the case of low prior knowledge, the narrative framework may provide a connectable mental structure for the new information, since there is no sufficient mental model of the factual content to build upon (Wolfe & Mienko, 2007). For the same reason, analogies are supposed to facilitate grasping new (health) concepts (Galesic & Garcia‐Retamero, 2013; Gentner & Smith, 2013). Like narratives, analogies are assumed to capture the recipients' attention (Van Stee, 2018). Enriching a narrative with an analogy that incorporates the content of the narration (a so‐called narrative analogy) attempts to draw on those advantages to convey scientific information more understandably and engagingly. Yet, narrative elements could draw attention away from scientific information (Alexander, 2019; Lehman et al., 2007), making knowledge transfer subject to the texts' design and how the factual content is embedded within the text.

Set against this theoretical background, we tested whether providing additional information to explain the mechanisms behind influenza vaccine effectiveness affects people's perceptions of influenza vaccination and enhances knowledge about and beyond the information given in a fact box (i.e. enhanced knowledge compromises basic facts that are also covered in the fact box and further facts that are only specified in the additional texts). We used a fact box as a strong and conservative control condition and compared it to two conditions that provided either an expository text or an additional narrative analogy in addition to the fact box. The two text conditions explained the relevance and consequences of seasonality for the vaccine formulation and effectiveness—either factual or as an analogy. We expected the following:

*Perceived effectiveness hypothesis*: Participants reading an additional text (fact box plus expository text or narrative analogy) will perceive influenza vaccine effectiveness to be higher than participants in the control condition (fact box only).

*Knowledge hypothesis*: Participants reading an additional text (fact box plus expository text or narrative analogy) will have enhanced influenza knowledge compared to participants in the control condition (fact box only).

In addition, we explored whether the narrative analogy is more likely than the expository text to enhance the perceived vaccine effectiveness and may lead to a higher knowledge gain due to the narratives' and analogies' capability to facilitate comprehension (Gentner & Smith, 2013; Mar et al., 2021). We further explored whether the subjective evaluations of the information materials differ between both texts and whether the provided influenza vaccine benefit–risk profile attracts more attention when it is accompanied by a narrative and an analogy that are both supposed to make the information more engaging (Dahlstrom, 2014; Van Stee, 2018).

We also explored whether the additional explanations affect the perceived risk of vaccination and the resulting vaccination intention, since a higher perceived vaccine effectiveness has been found to be associated with lower perceived vaccine risk and greater behavioural intentions (Felgendreff et al., 2023; Mostafapour et al., 2019; Ort & Fahr, 2018; Schulz & Hartung, 2021).

## METHODS

The preregistration (https://aspredicted.org/3fdx‐hmtx.pdf), materials, data sets, analysis scripts, and supplements are available via the Open Science Framework (OSF; osf.io/g4b67). The research obtained ethical clearance from the University IRB of Erfurt (approval number: 2023–14). All participants gave informed consent before data collection. If not otherwise stated, items were constructed for this study.

### Participants and design

Participants were stratified according to their response to a vaccine confidence item at the beginning of the survey and then randomly assigned to a one‐factorial between‐subjects design with three conditions: (1) fact box only (control), (2) fact box plus expository text or (3) fact box plus narrative analogy text. The stratified randomisation balanced potentially confounding pre‐existing vaccine beliefs as they can be expected to influence perceptions of vaccination information (Trevors, 2022). We used G*Power (Faul et al., 2007) for the power analysis and strived to reach a sample size of *N* = 1548 to detect a small effect in a one‐factorial analysis of variance (ANOVA) with three conditions (*f* = .1, *α* = .05, 1—*β* = .95). The German online panel GapFish recruited participants between 18 and 65 years of age during the last week of December 2022. Individuals could not participate if they had received an influenza vaccine that season, had participated in a previous study by the authors, or accessed the survey with a smartphone. The latter ensured that the fact box could be fully seen. Participants who failed the attention check described below (*n* = 132) were immediately screened out. One participant indicated being vaccinated against influenza at the survey's end and was excluded. The final data set included 1554 participants (for sociodemographic details, see Data S1).

### Materials and measures

#### Basic effectiveness information

**FIGURE 1:**
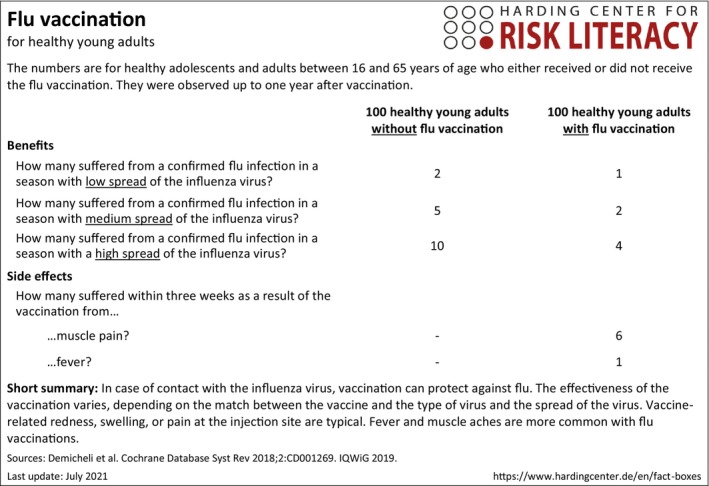
Fact box about flu vaccination as presented in the experiment (English Translation).

Each condition included the same fact box as described below. It provided data on the influenza vaccine's benefits and adverse events (Figure 1; Harding Center for Risk Literacy, 2021).

##### Fact box

The fact box compared the outcomes for a group of 100 unvaccinated people with those of a group of 100 vaccinated people. The potential benefits were fewer confirmed influenza cases than in the unvaccinated group for a season with (1) low, (2) medium and (3) widespread influenza virus. The potential adverse events concerned more vaccine reactions in the vaccinated group than in the unvaccinated group, that is, (1) more muscular soreness and (2) more fever in the following 3 weeks. A short statement within the fact box summarized the vaccine benefits and adverse events. Moreover, it pointed out that vaccination effectiveness varies depending on the spread of the virus and the match between the vaccine and the virus type. A short introduction (29 words) primed the reader in the fact box only condition that the fact box summarized scientific studies about how well the influenza vaccination can prevent the flu. This introduction obtained a readability index score (LIX) of 48.63 (Lenhard & Lenhard, 2011). Each condition received the fact box.

#### Additional explanations of effectiveness

The text conditions provided the fact box and additional explanations, as shown in Table 1 and detailed below.

**TABLE 1:** Excerpts of the information materials explaining core facts about the vaccination against influenza.

Core fact	Expository text	Narrative analogy
Seasonal character of circulating virus variants	Different virus variants of influenza are circulating worldwide. From year to year, which variants predominate in different regions change.	We can imagine flu viruses being like the bank robbers from the scenario described above. Just as Bernd is always expecting different robbers, our immune system is confronted with different flu viruses every flu season.
How the vaccination helps to prevent influenza	Vaccination prepares the immune system for the flu viruses expected during the season. The vaccine contains certain building blocks of the viral surface of these flu viruses. The immune system reacts to foreign building blocks. It forms immune cells that recognize these building blocks specifically and can destroy the viruses. (…) When real flu viruses enter the body later, the immune system recognizes the surface building blocks of the virus. Therefore, it fights the virus faster and in a more targeted manner. (…) Each season, the flu vaccines are adjusted to provide the best possible protection against current variants.	Vaccination trains the immune system before exposure to the flu viruses. A vaccine contains selected virus building blocks and thus transmits, so to speak, the ‘Wanted’ pictures of known pathogens. Each season, flu vaccines are adjusted to provide the best possible protection against current flu variants. This allows the immune system to fight these flu viruses more quickly and in a more targeted manner when they enter the body.
The relevance of the antigen–virus match	The virus building blocks in the current vaccine can be a very good match for the virus variants that occur during the season. In this case, the vaccine provides the best protection. It also happens that the virus components in the vaccination do not match the virus variants that occur that season so well. However, in such cases, the vaccination can still mitigate the course and complications of the flu. Existing protection from vaccinations from previous years also helps.	As with the ‘Wanted’ picture of the male bank robber, the vaccine may not match the flu virus exactly and may only provide an approximate heads‐up on the pathogen. Nevertheless, because the immune system has been put on alert, vaccination can mitigate the course and complications of the flu. Existing protection from previous years—Bernd's years of professional experience, so to speak—also helps.

*Note*: The full materials are provided at OSF (https://osf.io/g4b67).

#### Expository text

The expository text (275 words, LIX = 46.91) covered in simple terms the seasonal circulation of different variants of influenza viruses and how the vaccine prevents influenza. It explained that the vaccine's protection works best when the seasonal influenza vaccination matches the circulating virus variants well. If it does not match the variants perfectly, it could still prevent a severe course of influenza. The fact box and an additional summarizing sentence showed that vaccination can prevent about half of influenza cases. The expository text concluded that vaccination helps preventing influenza and a (severe) course of influenza.

#### Narrative analogy

The narrative analogy (501 words, LIX = 46.92) told a story about a security guard who was alerted about a potential bank robbery and used ‘Wanted’ pictures to prepare himself for a confrontation. The analogy to a ‘Wanted’ picture has been previously used to explain the vaccine mechanism (Coffee & Perkins, 2021). Here, the influenza virus was compared to a bank robber, the immune system to the bank security and the specific part of the virus in the vaccine to a ‘Wanted’ picture of the bank robber. As shown in Table 1, this analogy illustrated the expository text's core facts: (i) different bank robbers represent the seasonal character of circulating virus variants, (ii) the vaccination helps to prevent infections by providing a ‘Wanted’ picture, (iii) the relevance of the antigen–virus match is represented by a ‘Wanted’ picture not describing the actual influenza virus well, and (iv) the fact the even a mismatch alerts the bank security as it alerts the immune system. As in the expository text condition, the text concluded with the fact box and the final statement, as stated above.

#### Dependent variables

Participants stated their perceived vaccine effectiveness (‘What do you think: How effective is the flu shot in preventing the flu?’) on a seven‐point scale (1 = ‘not effective at all’ to 7 = ‘very effective’). Participants also indicated their perceived risk of vaccination (‘How risky do you think it would be for you to be vaccinated against flu?’) on a seven‐point scale (1 = ‘not risky at all’ to 7 = ‘very risky’). The vaccination intention (‘How would you decide if you had the opportunity next week to be vaccinated against flu?’) was introduced by the official German vaccination recommendation for influenza and rated on a seven‐point scale (1 = ‘no way vaccinate’ to 7 = ‘vaccinate in any case’) (all items adapted from Felgendreff et al., 2024). The knowledge test (low reliability: McDonald's *ω* = 0.55) was constructed based on the informative texts and comprised five single‐choice questions with five response options each. The basic knowledge questions covered content provided by the fact box: the type of pathogen, vaccine effectiveness (antigen–virus match) and vaccine side effects. The enhanced knowledge questions covered the vaccination schedule and the vaccine mechanism (how it works); this information was only provided in the two text conditions. Answers were coded as correct (1) or false (0) and aggregated to a mean score from 0 to 1, interpretable as relative proportion of correctly answered items. A g‐factor model tested the factorial validity of the enhanced knowledge construct (all five questions). All loadings were significant and the model displayed a good fit. The test demonstrated configural and metric invariance but not scalar invariance. Consequently, we also report item‐level analyses in the ‘Results’ section.

#### Confounding variable

The participants' influenza vaccination readiness was assessed with the 7C short scale (7 items; Geiger et al., 2021). It had sufficient reliability and validity (Geiger et al., 2021). The included item on vaccine confidence (‘I am convinced the appropriate authorities do only allow effective and safe flu vaccines’, 1 = ‘strongly disagree’ to 7 = ‘strongly agree’) was used for the stratified randomisation. Data S1 shows the successful balancing of vaccine confidence.

#### Attention check

The introduction informed the participants that incorrectly answering the attention check would result in immediate exclusion. Participants had to choose the age group mentioned in the fact box from five response options. The fact box was absent during the attention check.

#### Evaluation of material

Participants retrospectively rated the anger and fear they had experienced while reading the material on a seven‐point scale (adapted from Felgendreff et al., 2024). They also rated the material on several seven‐point scales regarding how appealing, well‐made, reliable, understandable, convincing and interesting they found the material (Rössler, 2011).

#### Attentional focus

Participants retrospectively stated on a seven‐point scale how much attention they had paid reading the fact box. For this, we adapted the ‘attentional focus’ subscale of the Narrative Engagement Scale (3 items; Busselle & Bilandzic, 2009). The attentional focus scale is an established measure with sufficient reliability and validity. In this study, the subscale yielded very good factorial validity, a perfect fit, and acceptable invariance. The three items' ratings were averaged (sufficient reliability: McDonald's *ω* = 0.90), with low scores indicating a high attentional focus.

#### Sociodemographic variables

Participants indicated their age, gender, highest level of school education and vaccination status (adapted from Felgendreff et al., 2024). They stated whether they met the listed criteria for an official vaccination recommendation by the German National Immunization Technical Advisory Group (STIKO; ‘yes,’ ‘no’) and whether they had been vaccinated against influenza in the past (‘yes,’ ‘no,’ ‘don't know’).

#### Further variables

Participants solved the Berlin Numeracy Test (single item; Cokely et al., 2012) and could comment on the survey in a text field. These variables are listed for transparency but are not analysed here.

### Procedure

The survey started with demographic information. Participants then read a comprehensive summary of the transmission and possible courses of influenza (asymptomatic, mild and severe) to provide them with key facts about influenza. Next, they indicated their vaccination readiness. Participants should then imagine an online search for information about influenza vaccination and were provided with the information materials according to their randomly assigned condition. The narrative analogy was split into two pages to reduce the need for scrolling. After the attention check, the information materials were displayed and remained visible when answering questions regarding their vaccine perceptions, vaccination intention, material evaluation questions and questions about attentional focus. Afterwards, participants completed the knowledge test and numeracy item and could leave comments about the study. Finally, the debriefing informed them about the research question and pointed to more information about influenza vaccination.

### Statistical analysis

The hypotheses were analysed using preregistered ANOVAs with Helmert contrast. The first Helmert contrast compared the fact box only condition with the two text conditions. The second Helmert contrast explored the difference between the expository text and the narrative analogy conditions. Cohen's *d* indicated the effect sizes for comparisons between the contrasts. Explorative one‐factorial ANOVAs with the perceived risk of vaccination and intention to get vaccinated as dependent variables were conducted with the same Helmert contrast. Simple one‐factorial ANOVAs explored the evaluation of the material and attentional focus. If not stated otherwise, nonparametric Kruskal–Wallis tests confirmed all results (see Data S1). Χ^*2*^‐test explored performance on knowledge items depending on condition; Cohen's *w* was used as effect size. Further exploration tested whether the material impacted the correlation between perceptions and intention using Fishers' *z*. A simple mediation analysis using structural equation modelling with bootstrapped standard errors and 95% confidence intervals of estimates (5000 iterations) explored whether the perceived effectiveness mediated the effect of the text interventions on intention.

## RESULTS

### Perceived vaccine effectiveness in preventing influenza

**FIGURE 2:**
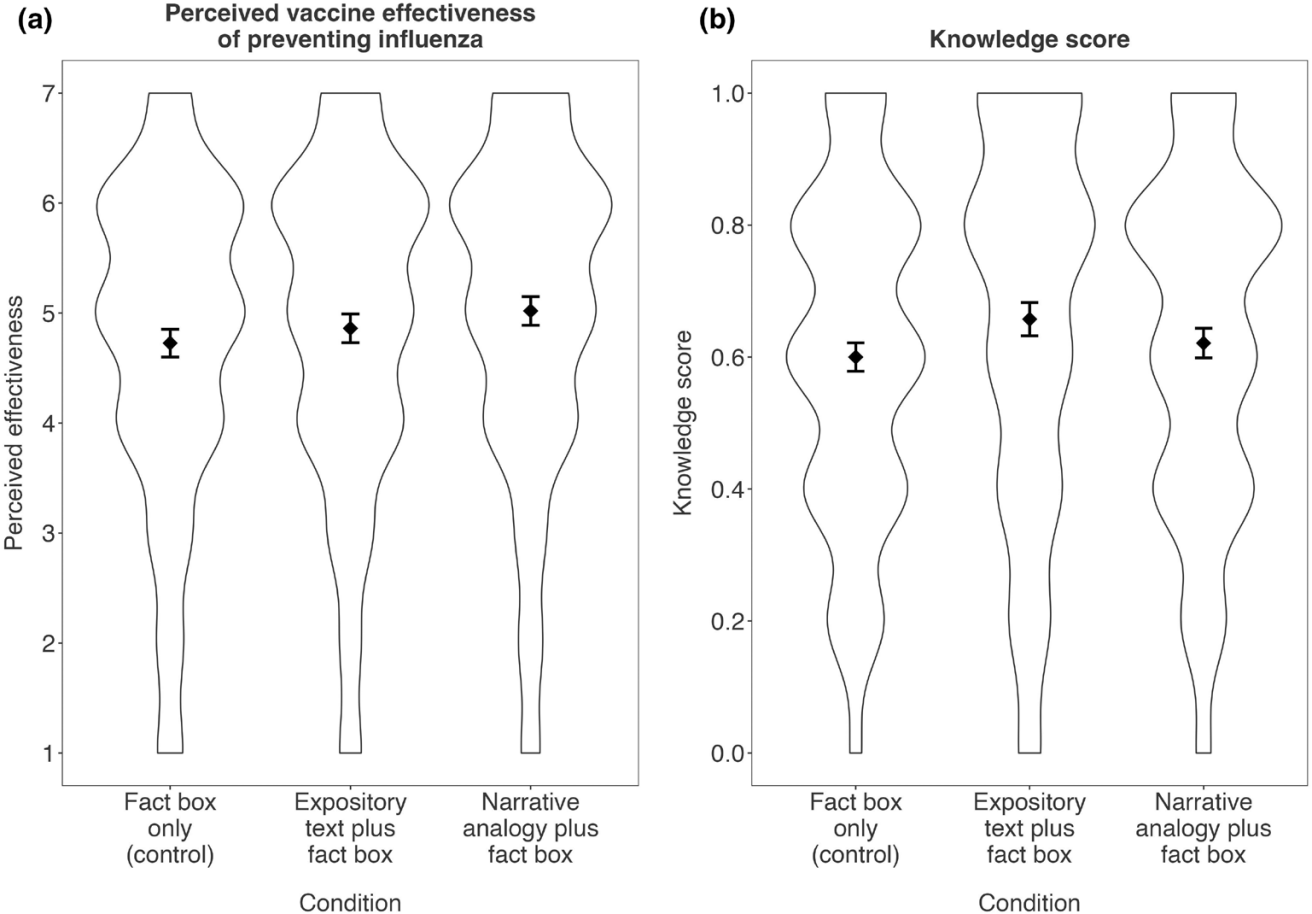
Means and distributions of perceived vaccine effectiveness and knowledge scores across conditions. The violin plots visualize the density distributions of the responses. The diamonds indicate group means and the whiskers represent the bootstrapped 95% confidence intervals. (a) An expository text or a narrative analogy increased the perceived influenza vaccine effectiveness as compared to the control condition (fact box only). (b) Participants in the expository text condition scored highest on the enhanced knowledge test (significant mean differences compared with fact box only and narrative analogy conditions).

Figure 2 illustrates the results of the protocol analyses for perceived effectiveness and enhanced knowledge. A one‐factorial ANOVA with a Helmert contrast between the control and text conditions showed that the mean perceived vaccine effectiveness to prevent influenza was higher in conditions with a text explaining the vaccine mechanism (*M* = 4.94, *SD* = 1.50, *n* = 1023) than in the condition displaying only the fact box (*M* = 4.73, *SD* = 1.49, *n* = 531; *F*(1, 1551) = 7.13, *p* = .008, $\eta^{2}$ <.01, *d* = −0.16). The results thus confirmed the perceived effectiveness hypothesis. The second Helmert contrast comparing the expository text and the narrative analogy did not show a difference (*F*(1,1551) = 2.87, *p* = .091, $\eta^{2}$ <.01, *d* = −0.11).

### Knowledge

The preregistered one‐factorial ANOVA with a Helmert contrast confirmed the knowledge hypothesis: the average enhanced knowledge score was higher in the text conditions (*M* = 0.64, *SD* = 0.28, *n* = 1023) than in the control condition (*M* = 0.60, *SD* = 0.25, *n* = 531; *F*(1, 1551) = 7.52, *p* = .006, $\eta^{2}$ < .01, *d* = −0.17). The second Helmert contrast showed that the expository text drove this effect: Participants in the expository condition (*M* = 0.66, *SD* = 0.29, *n* = 512) scored, on average, higher on the enhanced knowledge test than participants in the narrative analogy condition (*M* = 0.62, *SD* = 0.26, *n* = 511; *F*(1, 1545) = 4.69, *p* = .030, $\eta^{2}$ < .01, *d* = 0.13). Table 2 shows the results on item level (see Data S1 for descriptive statistics). When looking at the subset of the three single basic knowledge items (that assessed information provided in the fact box and thus in all conditions), the expository text group performed better than the fact box group on two of the three items, and the narrative analogy on one of the three items. Rerunning the ANOVA with basic knowledge as a dependent variable revealed no significant differences in the Helmert contrasts (all *F*s <2.54), albeit the Kruskal–Wallis tests indicated a difference between the text conditions and the control condition (*Χ* ^*2*^(1) = 5.11, *p* = .024), but not between the expository text and the narrative analogy (*Χ* ^*2*^(1) = 2.19, *p* = .139).

**TABLE 2:** Overview of information asked in the knowledge test, provided by the experimental material and the results for the group comparison of correct answers given (*N* = 1554).

Knowledge item on …	Information provided in the material			Group comparison of correct answers		
	Fact box only	Expository text	Narrative analogy	Fact box only—Expository text	Fact box only—Narrative analogy	Expository text—Narrative analogy
Basic knowledge						
Pathogen	Yes	Yes	Yes	Χ^*2*^(1) = 5.26, *p* = .022, *w* = 0.07	Χ^*2*^(1) = 0.06, *p* = .806, *w* < .01	Χ^*2*^(1) = 3.86, *p* < .050, *w* = 0.06
Vaccine effectiveness (antigen–virus match)	Yes	Yes	Yes	Χ^*2*^(1) = 7.45, *p* = .006, *w* = 0.09	Χ^*2*^(1) = 4.01, *p* = .045, *w* = 0.06	Χ^*2*^(1) = 0.43, *p* = .511, *w* = 0.02
Side effects	Yes	Yes	Yes	Χ^*2*^(1) = 1.88, *p* = .170, *w* = 0.04	Χ^*2*^(1) = 0.86, *p* = .353, *w* = 0.03	Χ^*2*^(1) = 0.13, *p* = .718, *w* = 0.01
Enhanced knowledge						
Vaccination schedule	No	Yes	Yes	Χ^*2*^(1) = 1.77, *p* = .183, *w* = 0.04	Χ^*2*^(1) = 2.62, *p* = .106, *w* = 0.05	Χ^*2*^(1) = 0.04, *p* = .834, *w* < 0.01
Vaccine mechanism	No	Yes	Yes	Χ^*2*^(1) = 19.87, *p* < .001, *w* = 0.14	Χ^*2*^(1) = 0.16, *p* = .692, *w* = 0.01	Χ^*2*^(1) = 15.65, *p* < .001, *w* = 0.13

*Note*: A Χ^2^‐test was conducted to test for differences in conditions and Cohen's *w* stated the effect size.

### Exploration

#### Perceived risk and intention to vaccinate

Explorative one‐factorial ANOVAs with Helmert contrasts and risk perception and intention as dependent variables revealed no significant differences between the text conditions and the fact box only condition, as well as between the expository text and the narrative analogy (risk perception: all *F*s <0.66; intention: all *F*s <1.43; see Data S1 for means). The provided material also did not influence the relationship between effectiveness perception–risk perception and effectiveness perception–intention, as the correlations did not differ across conditions (all Fisher's *z*s <1.33; Data S1). A simple mediation analysis revealed that the effect of the conditions with an additional text on intention was fully mediated by the effectiveness perception (indirect effect *B* = 0.038, *p* = .007; direct effect *B* = −0.038, *p* = .063; total effect *B* = −0.000, *p* = .997; Data S1).

#### Evaluation of material

A one‐factorial ANOVA explored elicited emotions and evaluation of the materials. Table 3 shows detailed results. The participants reported similarly low levels of experienced anger and fear in all three conditions and found the materials similarly understandable, convincing, and interesting. The narrative analogy was rated as more appealing and better made than the fact box only condition. The participants in the expository text condition judged the information material as more reliable than those in the fact box only condition. The narrative analogy and the expository text received quite similar ratings for the evaluation dimensions.

**TABLE 3:** Means and standard deviations for perceptions and attentional focus by information material.

	Condition						Main effect
	Fact box only		Expository text		Narrative analogy		
	M	SD	M	SD	M	SD	
Emotion							
Anger	2.18	1.56	2.14	1.57	2.24	1.64	*F*(2, 1551) = 0.57, *p* = .566, $\eta_{p}^{2}$ < .01
Fear	2.09	1.50	2.09	1.53	2.13	1.47	*F*(2, 1551) = 0.14, *p* = .872, $\eta_{p}^{2}$ < .01
Evaluation of material							
Appealing^a^	4.21	1.74	4.38	1.75	4.52	1.69	*F*(2, 1551) = 4.34, *p* = .013, $\eta_{p}^{2}$ < .01
Well‐made^a^	4.61	1.65	4.76	1.65	4.86	1.62	*F*(2, 1551) = 3.18, *p* = .042, $\eta_{p}^{2}$ < .01
Reliable^b^, ^c^	5.04	1.59	5.29	1.60	5.06	1.62	*F*(2, 1551) = 3.68, *p* = .025, $\eta_{p}^{2}$ < .01
Understandable	5.29	1.61	5.47	1.50	5.36	1.53	*F*(2, 1551) = 1.89, *p* = .152, $\eta_{p}^{2}$ < .01
Convincing	4.34	1.69	4.52	1.73	4.49	1.72	*F*(2, 1551) = 1.50, *p* = .223, $\eta_{p}^{2}$ < .01
Interesting	4.61	1.76	4.74	1.75	4.70	1.66	*F*(2, 1551) = 0.77, *p* = .463, $\eta_{p}^{2}$ < .01
Attentional focus (fact box)^d^	2.75	1.62	2.83	1.63	2.98	1.70	*F*(2, 1551) = 2.63, *p* = .073, $\eta_{p}^{2}$ < .01

*Note*: A one‐factorial ANOVA was conducted to test for differences between conditions. Attentional focus was inverse‐coded: high scores indicate low attentional focus when reading the fact box.

^a^ Post hoc test revealed that the narrative analogy condition's mean was significantly higher than the control condition's mean.

^b^ Post hoc test revealed that the expository text condition's mean was significantly higher than the control condition's mean.

^c^ Kruskal–Wallis test revealed that the expository text condition's median was significantly higher than the narrative analogy condition's median (H(1) = 6.18, *p* = .01).

^d^ Kruskal–Wallis test revealed that the control condition's median was significantly higher than the narrative analogy condition's median (H[1] = 5.05, *p* = .02).

#### Attentional focus

A one‐factorial ANOVA explored whether attentional focus while reading the fact box varied according to condition. The difference between the conditions was not significant (Table 3). However, the non‐parametric Kruskal–Wallis test indicated that the median attentional focus was higher when reading only the fact box than when also reading the narrative analogy (low scores indicate high attentional focus).

## DISCUSSION

Influenza vaccine effectiveness varies over the seasons and might evoke the impression that the vaccine is insufficient (Abu‐rish et al., 2016; Schmid et al., 2017; Seefeld et al., 2022) and, therefore, less desirable. Thus, understanding why vaccine effectiveness varies is crucial. This study presented effectiveness information in a state‐of‐the‐art way by showing participants a fact box. To enhance the knowledge, we supplemented the fact box with an expository text or a narrative analogy explaining why the seasonal influenza vaccine needs to be updated regularly, how it protects against an influenza disease, and that it can help even if the antigen–virus match is imperfect. As the *perceived effectiveness hypothesis* predicted, both texts somewhat increased the perceived vaccine effectiveness compared with only a fact box. Thus, providing relevant context information on why vaccine effectiveness can be limited can promote—instead of undermine—perceived vaccine effectiveness. This finding may generalize to further seasonal vaccines such as the vaccination against COVID. Supporting appropriate perceptions is a valuable part of an effective vaccine communication strategy (Ort & Fahr, 2018; Schulz & Hartung, 2021). Looking at the pattern of the single knowledge items, it seems that using enhancing information might also somewhat help to better understand some of the information provided in the fact box. Yet, it is important to state that the current study design does not allow conclusions regarding which fact influenced the perceived effectiveness. Further research could thus explore whether there are more or less important aspects in influencing appropriate perceptions. Fact boxes have been shown to foster vaccine benefits perceptions and vaccine confidence (Felgendreff et al., 2023; Rebitschek et al., 2022). Thus, additionally explaining vaccine effectiveness—for example, by having expository texts or narrative analogies address this aspect—may add to perceiving vaccination as effective, albeit to a limited extent.

We found that providing additional and more detailed information in a text led to enhanced knowledge. Reading either text increased the likelihood of identifying the antigen–virus match as critical for vaccine effectiveness. Yet, the expository text seemed more suitable than the narrative analogy for conveying certain basic and enhanced knowledge facts (Table 2). These results contrast several studies showing a similar knowledge gain in expository texts and narratives (Ecker et al., 2020; Flemming et al., 2018; Mensink et al., 2021; Wolfe & Mienko, 2007; Zebregs et al., 2015). It is possible that the narrative beginning of the analogy made the text less coherent, which could have led to impaired recall of the embedded scientific facts and, thus, lower knowledge scores (Mensink et al., 2021). Also, narrative elements could have drawn the attention away from the details of scientific explanations (Alexander, 2019; Lehman et al., 2007) so that participants only remembered the most prominently illustrated fact (the antigen–virus match).

Previous empirical findings suggest that perceiving greater vaccine effectiveness can be related to lower perceived vaccine risk and greater vaccination intention (Felgendreff et al., 2023; Mostafapour et al., 2019; Ort & Fahr, 2018; Schulz & Hartung, 2021). In this study, the perceived risk of vaccination against influenza and the vaccination intention were similar across conditions, as were their correlations with perceived effectiveness. Thus, while the additional texts strengthened the perception of the influenza vaccination as an effective protection strategy, they neither influenced the evaluation of the vaccination risks nor translated into a direct change in behavioural intentions. The perceived effectiveness fully mediated the effect of the text interventions on intention, and there was no direct effect of the intervention on intention. These results can also be interpreted in favour of enabling an informed decision, since the additional explanations did not exert persuasive effects on non‐targeted beliefs or behavioural intentions. Low ratings on experienced anger and fear in all conditions back up this interpretation (low reactance; Brehm, 1966).

Despite the less detailed knowledge about single facts after receiving the narrative analogy, the similar perceived vaccine effectiveness in both text conditions suggests that metacognitive, non‐epistemic cues such as processing fluency might play a role (Schwarz, 2004). Research shows that the ease with which new information is processed mediates the relationship between provided information and outcomes of perception (Bullock & Shulman, 2021). The narrative analogy, rated as more appealing and better made than the fact box by itself, likely facilitated a more effortless processing of the information, thereby enhancing perceived effectiveness compared to the fact box only. Thus, although the narrative analogy did not promote the immediate memory of specific facts like the expository text, it could have enhanced the inferential ‘gist’‐understanding of vaccine effectiveness (Clinton et al., 2020; Reyna, 2021). This is also supported by the pattern presented in Table 2, suggesting that both texts also improved the basic knowledge on vaccine effectiveness (antigen–virus match). Hence, when using an analogy, the potential distraction of the reader from details needs to be kept in mind—while it may support understanding the gist, it may blur the single facts (Donnelly & McDaniel, 1993). These potential different learning outcomes should be considered when using narrative analogies in health communication.

### Limitations

Some limitations need to be considered when interpreting the results. Overall, the obtained effect sizes were small, possibly because the texts did not persuasively emphasize the personal vaccine benefits and the fact box provided vaccine benefit information in a numeric way in all conditions. Next, the study implemented a post‐treatment design. While we balanced a priori vaccine confidence with a stratified sampling procedure, the distribution of pre‐treatment knowledge is unknown. Future studies could test how prior knowledge influences the relationship between the different text formats and enhanced knowledge (Dong et al., 2020; Hailikari et al., 2008; Wolfe & Mienko, 2007). In addition, the reliability of the knowledge test was not sufficient. The low reliability was driven by the knowledge item about the vaccine mechanism, which was very difficult (see Data S1). We also reported the results at the item level to address this limitation. As for the knowledge test, some of the measures were created for the study and have not been validated before this study. Where available, we reported reliability and validity information in the Methods section and conducted validity tests where possible. Finally, the two texts varied in length. It is possible that the greater length of the narrative analogy led to less thorough reading (especially in the context of an online experiment), and, therefore, the effects on knowledge may be treated with care.

## CONCLUSION

Without a universal influenza vaccine covering all potential new strains, the seasonal influenza vaccine's effectiveness will always depend on how well the circulating influenza strains match the given vaccine formulation (WHO, 2022). Thus, uncertainty about the influenza vaccines' effectiveness in preventing influenza disease is, to some extent, justified and contributes to people omitting seasonal vaccinations (Schmid et al., 2017). Our study showed that explaining why the seasonal influenza vaccine needs to be updated regularly and how it helps protect against influenza disease, even if the antigen–virus match is imperfect, can increase effectiveness perceptions.

## AUTHOR CONTRIBUTIONS

**Lisa Felgendreff:** Conceptualization; investigation; methodology; writing – original draft; writing – review and editing; formal analysis. **Felix G. Rebitschek:** Funding acquisition; conceptualization; writing – review and editing. **Parichehr Shamsrizi:** Writing – review and editing; conceptualization. **Mattis Geiger:** Writing – review and editing; formal analysis. **Mirjam A. Jenny:** Funding acquisition; writing – review and editing. **Cornelia Betsch:** Writing – review and editing; conceptualization.

## Supporting information


**DataS1**.

## Data Availability

The data that support the findings of this study are openly available in Open Science Framework at https://osf.io/g4b67.

## References

[bjhp12770-bib-0001] Abu‐rish, E. Y. , Elayeh, E. R. , Mousa, L. A. , Butanji, Y. K. , & Albsoul‐Younes, A. M. (2016). Knowledge, awareness and practices towards seasonal influenza and its vaccine: Implications for future vaccination campaigns in Jordan. Family Practice, 33(6), 690–697. 10.1093/fampra/cmw086 27567011 PMC7188315

[bjhp12770-bib-0002] Alexander, P. A. (2019). The art (and science) of seduction: Why, when, and for whom seductive details matter. Applied Cognitive Psychology, 33(1), 142–148. 10.1002/acp.3510

[bjhp12770-bib-0003] Brehm, J. W. (1966). A theory of psychological reactance. Academic Press.

[bjhp12770-bib-0004] Brick, C. , McDowell, M. , & Freeman, A. L. J. (2020). Risk communication in tables versus text: A registered report randomized trial on ‘fact boxes’. Royal Society open Science, 7(3), 190,876. 10.1098/rsos.190876 PMC7137953 32269779

[bjhp12770-bib-0005] Bullock, O. M. , & Shulman, H. C. (2021). Utilizing framing theory to design more effective health messages about tanning behavior among college women. Communication Studies, 72(3), 319–332. 10.1080/10510974.2021.1899007

[bjhp12770-bib-0006] Busselle, R. , & Bilandzic, H. (2009). Measuring narrative engagement. Media Psychology, 12(4), 321–347. 10.1080/15213260903287259

[bjhp12770-bib-0007] Cameron, K. A. , Roloff, M. E. , Friesema, E. M. , Brown, T. , Jovanovic, B. D. , Hauber, S. , & Baker, D. W. (2013). Patient knowledge and recall of health information following exposure to "facts and myths" message format variations. Patient Education and Counseling, 92(3), 381–387. 10.1016/j.pec.2013.06.017 23891420 PMC3772650

[bjhp12770-bib-0008] Clinton, V. , Taylor, T. , Bajpayee, S. , Davison, M. L. , Carlson, S. E. , & Seipel, B. (2020). Inferential comprehension differences between narrative and expository texts: A systematic review and meta‐analysis. Reading and Writing, 33(9), 2223–2248. 10.1007/s11145-020-10044-2

[bjhp12770-bib-0009] Coffee, M. , & Perkins, S. (2021). Vaccines for dummies (1st ed.). John Wiley and Sons.

[bjhp12770-bib-0010] Cokely, E. T. , Galesic, M. , Schulz, E. , Ghazal, S. , & Garcia‐Retamero, R. (2012). Measuring risk literacy: The Berlin numeracy test. Judgment and Decision Making, 7(1), 25–47.

[bjhp12770-bib-0011] Council of the European Union . (2009). Council recommendation of 22 December 2009 on seasonal influenza vaccination (text with EEA relevance) (2009/1019/EU). Official Journal of the European Union, L348, 71–72. https://eur‐lex.europa.eu/LexUriServ/LexUriServ.do?uri=OJ:L:2009:348:0071:0072:EN:PDF

[bjhp12770-bib-0012] Dahlstrom, M. F. (2014). Using narratives and storytelling to communicate science with nonexpert audiences. Proceedings of the National Academy of Sciences, 111(supplement_4), 13,614–13,620. 10.1073/pnas.1320645111 PMC4183170 25225368

[bjhp12770-bib-0013] Demicheli, V. , Jefferson, T. , Ferroni, E. , Rivetti, A. , & Di Pietrantonj, C. (2018). Vaccines for preventing influenza in healthy adults. Cochrane Database of Systematic Reviews, 2020(2), CD001269. 10.1002/14651858.CD001269.pub6 PMC6491184 29388196

[bjhp12770-bib-0014] Dong, A. , Jong, M. S.‐Y. , & King, R. B. (2020). How does prior knowledge influence learning engagement? The mediating roles of cognitive load and help‐seeking. Frontiers in Psychology, 11(591), 203. 10.3389/fpsyg.2020.591203 33192933 PMC7658369

[bjhp12770-bib-0015] Donnelly, C. M. , & McDaniel, M. A. (1993). Use of analogy in learning scientific concepts. Journal of Experimental Psychology: Learning, Memory, and Cognition, 19(4), 975–987. 10.1037/0278-7393.19.4.975 8345330

[bjhp12770-bib-0016] Ecker, U. K. H. , Butler, L. H. , & Hamby, A. (2020). You don't have to tell a story! A registered report testing the effectiveness of narrative versus non‐narrative misinformation corrections. Cognitive Research: Principles and Implications, 5(1), 64. 10.1186/s41235-020-00266-x 33300094 PMC7725032

[bjhp12770-bib-0017] Faul, F. , Erdfelder, E. , Lang, A.‐G. , & Buchner, A. (2007). G*power 3: A flexible statistical power analysis program for the social, behavioral, and biomedical sciences. Behavior Research Methods, 39(2), 175–191. 17695343 10.3758/bf03193146

[bjhp12770-bib-0018] Felgendreff, L. , Rebitschek, F. G. , Geiger, M. , Shamsrizi, P. , Jenny, M. A. , & Betsch, C. (2024). Explaining the mechanism behind mRNA vaccines influences perceived vaccine effectiveness but not vaccination intentions: A randomized experiment. European Journal of Health Communication, 5(1), 21–45. 10.47368/ejhc.2024.102.

[bjhp12770-bib-0019] Felgendreff, L. , Renkewitz, F. , & Betsch, C. (2023). How communicating vaccine benefits and harms in fact boxes affects risk perceptions: Two randomized trials. European. Journal of Health Psychology, 30, 2512‐8442/a000134. 10.1027/2512-8442/a000134

[bjhp12770-bib-0020] Flemming, D. , Cress, U. , Kimmig, S. , Brandt, M. , & Kimmerle, J. (2018). Emotionalization in science communication: The impact of narratives and visual representations on knowledge gain and risk perception. Frontiers in Communication, 3, 3. 10.3389/fcomm.2018.00003

[bjhp12770-bib-0021] Galesic, M. , & Garcia‐Retamero, R. (2013). Using analogies to communicate information about health risks: Analogies for health communication. Applied Cognitive Psychology, 27(1), 33–42. 10.1002/acp.2866

[bjhp12770-bib-0022] Geiger, M. , Rees, F. , Lilleholt, L. , Santana, A. P. , Zettler, I. , Wilhelm, O. , Betsch, C. , & Böhm, R. (2021). Measuring the 7Cs of vaccination readiness. European Journal of Psychological Assessment, 1–9, 261–269. 10.1027/1015-5759/a000663

[bjhp12770-bib-0023] Gentner, D. , & Smith, L. A. (2013). Analogical learning and reasoning. In Reisber, D. (Ed.), The Oxford Handbook of Cognitive Psychology, Oxford Library of Psychology (2023; online edn, Oxford Academic, 3 June 2013). 10.1093/oxfordhb/9780195376746.013.0042

[bjhp12770-bib-0024] Golke, S. , Hagen, R. , & Wittwer, J. (2019). Lost in narrative? The effect of informative narratives on text comprehension and metacomprehension accuracy. Learning and Instruction, 60, 1–19. 10.1016/j.learninstruc.2018.11.003

[bjhp12770-bib-0025] Gravagna, K. , Wolfson, C. , Sulis, G. , Buchan, S. A. , McNeil, S. , Andrew, M. K. , McMillan, J. , Kirkland, S. , & Basta, N. E. (2022). Influenza vaccine coverage and factors associated with non‐vaccination among adults at high risk for severe outcomes: An analysis of the Canadian longitudinal study on aging. PLoS One, 17(9), e0275135. 10.1371/journal.pone.0275135 36178943 PMC9524702

[bjhp12770-bib-0026] Hailikari, T. , Katajavuori, N. , & Lindblom‐Ylanne, S. (2008). The relevance of prior knowledge in learning and instructional design. American Journal of Pharmaceutical Education, 72(5), 113. 10.5688/aj7205113 19214267 PMC2630138

[bjhp12770-bib-0027] Harding Center for Risk Literacy . (2021). Grippeschutzimpfung für Erwachsene (Influenzaimpfung) [Influenza vaccination for adults (influenza vaccination)]. Harding‐Zentrum Für Risikokompetenz https://www.hardingcenter.de/de/transfer‐und‐nutzen/faktenboxen/impfungen/grippeschutzimpfung‐fuer‐erwachsene‐influenzaimpfung.

[bjhp12770-bib-0028] Hinyard, L. J. , & Kreuter, M. W. (2007). Using narrative communication as a tool for health behavior change: A conceptual, theoretical, and empirical overview. Health Education & Behavior, 34(5), 777–792. 10.1177/1090198106291963 17200094

[bjhp12770-bib-0029] Iuliano, A. D. , Roguski, K. M. , Chang, H. H. , Muscatello, D. J. , Palekar, R. , Tempia, S. , Cohen, C. , Gran, J. M. , Schanzer, D. , Cowling, B. J. , Wu, P. , Kyncl, J. , Ang, L. W. , Park, M. , Redlberger‐Fritz, M. , Yu, H. , Espenhain, L. , Krishnan, A. , Emukule, G. , … Mustaquim, D. (2018). Estimates of global seasonal influenza‐associated respiratory mortality: A modelling study. The Lancet, 391(10127), 1285–1300. 10.1016/S0140-6736(17)33293-2 PMC5935243 29248255

[bjhp12770-bib-0030] Lehman, S. , Schraw, G. , McCrudden, M. T. , & Hartley, K. (2007). Processing and recall of seductive details in scientific text. Contemporary Educational Psychology, 32(4), 569–587. 10.1016/j.cedpsych.2006.07.002

[bjhp12770-bib-0031] Lenhard, W. , & Lenhard, A. (2011). Berechnung des Lesbarkeitsindex LIX nach Björnson [calculation of the readability index LIX according to Björnson]. 10.13140/RG.2.1.1512.3447

[bjhp12770-bib-0032] Lühnen, J. , Albrecht, M. , Hanßen, K. , Hildebrandt, J. , & Steckelberg, A. (2015). Leitlinie evidenzbasierte Gesundheitsinformation: Einblick in die Methodik der Entwicklung und Implementierung [guideline for the development of evidence‐based patient information: Insights into the methods and implementation of evidence‐based health information]. Zeitschrift für Evidenz, Fortbildung Und Qualität Im Gesundheitswesen, 109(2), 159–165. 10.1016/j.zefq.2015.03.004 26028454

[bjhp12770-bib-0033] Lühnen, J. , Albrecht, M. , Mühlhauser, I. , & Steckelberg, A. (2017). Leitlinie evidenzbasierte Gesundheitsinformation. http://www.leitlinie‐gesundheitsinformation.de/wp‐content/uploads/2017/07/Leitlinie‐evidenzbasierte‐Gesundheitsinformation.pdf 10.1016/j.zefq.2015.03.004 26028454

[bjhp12770-bib-0034] Macias, A. E. , McElhaney, J. E. , Chaves, S. S. , Nealon, J. , Nunes, M. C. , Samson, S. I. , Seet, B. T. , Weinke, T. , & Yu, H. (2021). The disease burden of influenza beyond respiratory illness. Vaccine, 39, A6–A14. 10.1016/j.vaccine.2020.09.048 33041103 PMC7545338

[bjhp12770-bib-0035] Maddux, J. E. , & Rogers, R. W. (1983). Protection motivation and self‐efficacy: A revised theory of fear appeals and attitude change. Journal of Experimental Social Psychology, 19(5), 469–479. 10.1016/0022-1031(83)90023-9

[bjhp12770-bib-0036] Mar, R. A. , Li, J. , Nguyen, A. T. P. , & Ta, C. P. (2021). Memory and comprehension of narrative versus expository texts: A meta‐analysis. Psychonomic Bulletin & Review, 28(3), 732–749. 10.3758/s13423-020-01853-1 33410100 PMC8219577

[bjhp12770-bib-0037] McDowell, M. , Gigerenzer, G. , Wegwarth, O. , & Rebitschek, F. G. (2019). Effect of tabular and icon fact box formats on comprehension of benefits and harms of prostate cancer screening: A randomized trial. Medical Decision Making, 39(1), 41–56. 10.1177/0272989X18818166 30799691

[bjhp12770-bib-0038] McDowell, M. , Rebitschek, F. G. , Gigerenzer, G. , & Wegwarth, O. (2016). A simple tool for communicating the benefits and harms of health interventions: A guide for creating a fact box. MDM Policy & Practice, 1(1), 238,146,831,666,536. 10.1177/2381468316665365 PMC6125040 30288405

[bjhp12770-bib-0039] Mensink, M. C. , Kendeou, P. , & Rapp, D. N. (2021). Do different kinds of introductions influence comprehension and memory for scientific explanations? Discourse Processes, 58(5–6), 491–512. 10.1080/0163853X.2021.1904754

[bjhp12770-bib-0040] Meyer, S. B. , Lu, S. K. , Hoffman‐Goetz, L. , Smale, B. , MacDougall, H. , & Pearce, A. R. (2016). A content analysis of newspaper coverage of the seasonal flu vaccine in Ontario, Canada, October 2001 to march 2011. Journal of Health Communication, 21(10), 1088–1097. 10.1080/10810730.2016.1222038 27668454

[bjhp12770-bib-0041] Mitchell, G. , Leonard, L. , Carter, G. , Santin, O. , & Brown Wilson, C. (2021). Evaluation of a ‘serious game’ on nursing student knowledge and uptake of influenza vaccination. PLoS One, 16(1), e0245389. 10.1371/journal.pone.0245389 33444348 PMC7808644

[bjhp12770-bib-0042] Morales, K. F. , Brown, D. W. , Dumolard, L. , Steulet, C. , Vilajeliu, A. , Ropero Alvarez, A. M. , Moen, A. , Friede, M. , & Lambach, P. (2021). Seasonal influenza vaccination policies in the 194 WHO member states: The evolution of global influenza pandemic preparedness and the challenge of sustaining equitable vaccine access. Vaccine, X, 8, 100,097. 10.1016/j.jvacx.2021.100097 PMC8143996 34041476

[bjhp12770-bib-0043] Mostafapour, M. , Meyer, S. B. , & Scholer, A. (2019). Exploring the effect of risk and benefit information provision on vaccination decision‐making. Vaccine, 37(44), 6750–6759. 10.1016/j.vaccine.2019.08.083 31558328

[bjhp12770-bib-0044] Murdoch, B. , & Caulfield, T. (2018). Influenza vaccination discourse in major Canadian news media, 2017–2018. Heliyon, 4(11), e00970. 10.1016/j.heliyon.2018.e00970 30519662 PMC6260240

[bjhp12770-bib-0045] Neufeind, J. , Wenchel, R. , Boedeker, B. , Wicker, S. , & Wichmann, O. (2021). Monitoring influenza vaccination coverage and acceptance among health‐care workers in German hospitals—Results from three seasons. Human Vaccines & Immunotherapeutics, 17(3), 664–672. 10.1080/21645515.2020.1801072 33124954 PMC7993141

[bjhp12770-bib-0046] Ort, A. , & Fahr, A. (2018). Using efficacy cues in persuasive health communication is more effective than employing threats—An experimental study of a vaccination intervention against Ebola. British Journal of Health Psychology, 23(3), 665–684. 10.1111/bjhp.12310 29635864

[bjhp12770-bib-0047] Peasah, S. K. , Azziz‐Baumgartner, E. , Breese, J. , Meltzer, M. I. , & Widdowson, M.‐A. (2013). Influenza cost and cost‐effectiveness studies globally—A review. Vaccine, 31(46), 5339–5348. 10.1016/j.vaccine.2013.09.013 24055351

[bjhp12770-bib-0048] Rebitschek, F. G. , Ellermann, C. , Jenny, M. A. , Siegel, N. A. , Spinner, C. , & Wagner, G. G. (2022). Fact boxes that inform individual decisions may contribute to a more positive evaluation of COVID‐19 vaccinations at the population level. PLoS One, 17(9), e0274186. 10.1371/journal.pone.0274186 36095020 PMC9467356

[bjhp12770-bib-0049] Reyna, V. F. (2021). A scientific theory of gist communication and misinformation resistance, with implications for health, education, and policy. Proceedings of the National Academy of Sciences, 118(15), e1912441117. 10.1073/pnas.1912441117 PMC8054009 32312815

[bjhp12770-bib-0050] Rosenstock, I. M. (1974). Historical origins of the health belief model. Health Education Monographs, 2(4), 328–335. 10.1177/109019817400200403 299611

[bjhp12770-bib-0051] Rössler, P. (2011). Skalenhandbuch Kommunikationswissenschaft. VS Verlag für *Sozialwissenschaften*.

[bjhp12770-bib-0052] Schmid, P. , Rauber, D. , Betsch, C. , Lidolt, G. , & Denker, M.‐L. (2017). Barriers of influenza vaccination intention and behavior—A systematic review of influenza vaccine hesitancy, 2005–2016. PLoS One, 12(1), e0170550. 10.1371/journal.pone.0170550 28125629 PMC5268454

[bjhp12770-bib-0053] Schulz, P. J. , & Hartung, U. (2021). Unsusceptible to social communication? The fixture of the factors predicting decisions on different vaccinations. Health Communication, 36(12), 1505–1513. 10.1080/10410236.2020.1771119 32522030

[bjhp12770-bib-0054] Schwarz, N. (2004). Metacognitive experiences in consumer judgment and decision making. Journal of Consumer Psychology, 14(4), 332–348. 10.1207/s15327663jcp1404_2

[bjhp12770-bib-0055] Seefeld, L. , Horstkötter, N. , Ommen, O. , Reckendrees, B. , Stander, V. , Goecke, M. , Dietrich, M. , Müller, U. , & Leicht, J. (2022). Einstellungen, Wissen und Verhalten von Erwachsenen und Eltern gegenüber Impfungen—Ergebnisse der Repräsentativbefragung 2021 zum Infektionsschutz. Leitbegriffe der Gesundheitsförderung Und Prävention. 10.17623/BZGA:T2-IFSS-2021

[bjhp12770-bib-0056] Sheikh, S. , Biundo, E. , Courcier, S. , Damm, O. , Launay, O. , Maes, E. , Marcos, C. , Matthews, S. , Meijer, C. , Poscia, A. , Postma, M. , Saka, O. , Szucs, T. , & Begg, N. (2018). A report on the status of vaccination in Europe. Vaccine, 36(33), 4979–4992. 10.1016/j.vaccine.2018.06.044 30037416

[bjhp12770-bib-0057] Trevors, G. J. (2022). The roles of identity conflict, emotion, and threat in learning from refutation texts on vaccination and immigration. Discourse Processes, 59(1–2), 36–51. 10.1080/0163853X.2021.1917950

[bjhp12770-bib-0058] Troeger, C. E. , Blacker, B. F. , Khalil, I. A. , Zimsen, S. R. M. , Albertson, S. B. , Abate, D. , Abdela, J. , Adhikari, T. B. , Aghayan, S. A. , Agrawal, S. , Ahmadi, A. , Aichour, A. N. , Aichour, I. , Aichour, M. T. E. , Al‐Eyadhy, A. , Al‐Raddadi, R. M. , Alahdab, F. , Alene, K. A. , Aljunid, S. M. , … Reiner, R. C. (2019). Mortality, morbidity, and hospitalisations due to influenza lower respiratory tract infections, 2017: An analysis for the global burden of disease study 2017. The Lancet Respiratory Medicine, 7(1), 69–89. 10.1016/S2213-2600(18)30496-X 30553848 PMC6302221

[bjhp12770-bib-0059] Van Stee, S. K. (2018). Meta‐analysis of the persuasive effects of metaphorical vs. Literal Messages. Communication Studies, 69(5), 545–566. 10.1080/10510974.2018.1457553

[bjhp12770-bib-0060] Wolfe, M. B. W. , & Mienko, J. A. (2007). Learning and memory of factual content from narrative and expository text. British Journal of Educational Psychology, 77(3), 541–564. 10.1348/000709906X143902 17908374

[bjhp12770-bib-0061] Wolfe, M. B. W. , & Woodwyk, J. M. (2010). Processing and memory of information presented in narrative or expository texts. British Journal of Educational Psychology, 80(3), 341–362. 10.1348/000709910X485700 20128958

[bjhp12770-bib-0062] World Health Organization . (2022). Vaccines against influenza: WHO position paper—May 2022. Weekly Epidemiological Record, 97(19), 185–208.

[bjhp12770-bib-0063] Zebregs, S. , van den Putte, B. , de Graaf, A. , Lammers, J. , & Neijens, P. (2015). The effects of narrative versus non‐narrative information in school health education about alcohol drinking for low educated adolescents. BMC Public Health, 15(1), 1085. 10.1186/s12889-015-2425-7 26499061 PMC4619486

