# Supplementary material for: Explaining seasonality increases perceived effectiveness of influenza vaccination: An experimental study
Source: Br J Health Psychol. 2024 Nov 28;30(1):e12770. doi: 10.1111/bjhp.12770 (PMC11604028; doi:10.1111/bjhp.12770)
Supplement: Supplementary file 1 — DataS1. [file BJHP-30-0-s001.docx]

# Supplement

## Supplement S1

**Table S.1**

*Sociodemographic Details for the Final Sample (N = 1554)*

| Variable |  | Total |  | Condition |  | Main Effect |
| --- | --- | --- | --- | --- | --- | --- |
|  |  |  | Fact Box Only | Expository Text | Narrative Analogy |  |
| **N** | *n* (%) | 1554 (100.00) | 531 (34.17) | 512 (33.95) | 511 (32.88) | N/A |
| **Age** | *M (SD)* | 41.61 (13.30) | 41.23 (13.45) | 42.26 (12.89) | 41.34 (13.54) | *F*(2, 1551) = 0.93, *p* = .395, $\eta_{p}^{2}$ < .01 |
| **Gender** |  |  |  |  |  | Χ*^2^*(4) = 4.56,  *p* = .336 |
| Male | *n* (%) | 881 (56.69) | 310 (58.38) | 278 (54.30) | 293 (57.34) |  |
| Female | *n* (%) | 668 (43.99) | 219 (41.24) | 231 (45.12) | 218 (42.66) |  |
| Diverse | *n* (%) | 5 (0.32) | 2 (0.38) | 3 (0.59) | 0 (0.00) |  |
| **School education** |  |  |  |  |  | Χ*^2^*(10) = 15.01,  *p* = .132 |
| No school leaving certificate | *n* (%) | 9 (0.58) | 1 (0.19) | 5 (0.98) | 3 (0.59) |  |
| Lower track | *n* (%) | 133 (8.56) | 46 (8.66) | 44 (8.59) | 43 (8.41) |  |
| Intermediate track | *n* (%) | 510 (32.82) | 175 (32.96) | 181 (35.35) | 154 (30.14) |  |
| FH ^a^ entrance qualification | *n* (%) | 200 (12.87) | 72 (13.56) | 63 (12.30) | 65 (12.72) |  |
| University entrance qualification | *n* (%) | 687 (44.21) | 236 (44.44) | 215 (41.99) | 236 (46.18) |  |
| Other school type | *n* (%) | 15 (0.97) | 1 (0.19) | 4 (0.78) | 10 (1.96) |  |
| **Target group**  **of STIKO recommendation** |  |  |  |  |  | Χ*^2^*(2) = 4.94,  *p* = .085 |
| Yes | *n* (%) | 484 (31.15) | 184 (34.65) | 146 (28.52) | 154 (30.14) |  |
| No | *n* (%) | 1070 (68.85) | 347 (65.35) | 366 (71.48) | 357 (69.86) |  |
| **Past influenza vaccination** |  |  |  |  |  | Χ*^2^*(4) = 10.39,  *p* = .034 |
| Yes | *n* (%) | 712 (45.82) | 257 (48.40) | 233 (45.51) | 222 (43.44) |  |
| No | *n* (%) | 769 (49.49) | 240 (45.20) | 257 (50.20) | 272 (53.23) |  |
| Don’t know | *n* (%) | 73 (4.70) | 34 (6.40) | 22 (4.30) | 17 (3.33) |  |
| **7C short scale** |  |  |  |  |  |  |
| Mean score | *M (SD)* | 3.58 (1.10) | 3.56 (1.08) | 3.57 (1.11) | 3.60 (1.11) | *F*(2, 1551) = 0.24, *p* = .790, $\eta_{p}^{2}$ < .01 |
| Confidence | *M (SD)* | 4.62 (1.93) | 4.6 (1.94) | 4.6 (1.94) | 4.65 (1.92) | *F*(2, 1551) = 0.11, *p* = .897, $\eta_{p}^{2}$ < .01 |
| Complacency | *M (SD)* | 3.22 (1.92) | 3.17 (1.86) | 3.23 (1.99) | 3.27 (1.91) | *F*(2, 1551) = 0.37, *p* = .694, $\eta_{p}^{2}$ < .01 |
| Constraints | *M (SD)* | 2.68 (1.78) | 2.62 (1.73) | 2.71 (1.82) | 2.72 (1.81) | *F*(2, 1551) = 0.46, *p* = .631, $\eta_{p}^{2}$ < .01 |
| Calculation *(not reversed)* | *M (SD)* | 4.40 (1.92) | 4.45 (1.88) | 4.34 (1.94) | 4.42 (1.94) | *F*(2, 1551) = 0.44, *p* = .642, $\eta_{p}^{2}$ < .01 |
| Collective responsibility | *M (SD)* | 3.71 (1.93) | 3.75 (1.91) | 3.63 (1.92) | 3.73 (1.97) | *F*(2, 1551) = 0.52, *p* = .594, $\eta_{p}^{2}$ < .01 |
| Compliance | *M (SD)* | 2.13 (1.65) | 2.1 (1.58) | 2.14 (1.70) | 2.16 (1.67) | *F*(2, 1551) = 0.22, *p* = .805, $\eta_{p}^{2}$ < .01 |
| Conspiracy *(not reversed)* | *M (SD)* | 2.90 (1.74) | 2.87 (1.73) | 2.96 (1.75) | 2.87 (1.74) | *F*(2, 1551) = 0.41, *p* = .659, $\eta_{p}^{2}$ < .01 |
| **Numeracy item** |  |  |  |  |  | Χ*^2^*(2) = 0.20,  *p* = .904 |
| False | *n* (%) | 1200 (77.22) | 409 (77.02) | 393 (76.76) | 398 (77.89) |  |
| Correct | *n* (%) | 354 (22.78) | 122 (23.98) | 119 (23.24) | 113 (22.11) |  |

*Note.* A one-factorial ANOVA (metric variable level) or Χ*^2^*-test (nominal variable level) was conducted to test for differences between factor levels. N/A implies that comparison was not possible (only one group).

^a^ FH is the abbreviation for the German equivalent of ‘University for applied science’.

## Supplement S2

**Table S.2**

*Results of Nonparametric Kruskal–Wallis Test for Dependent Variables*

| Variable | Condition | | | |
| --- | --- | --- | --- | --- |
|  | Contrast 1:  Control vs Text | | Contrast 2:  Expository Text vs Narrative Analogy | |
|  | *H*(1) | *p* | *H*(1) | *p* |
| **Main dependent variables** |  |  |  |  |
| Perceived vaccine effectiveness of preventing Influenza | 8.35 | .004 | 3.47 | .063 |
| Knowledge test | 10.89 | < .001 | 7.57 | .006 |
| **Other dependent variables** |  |  |  |  |
| Perceived risk of vaccination | 0.81 | .367 | 0.04 | .850 |
| Intention to get vaccinated | < 0.01 | .981 | 1.44 | .231 |

**Table S.3**

*Results of Nonparametric Kruskal–Wallis Test for Explorative Variables*

| Variable |  | | Condition | | | | | |
| --- | --- | --- | --- | --- | --- | --- | --- | --- |
|  | Overall | | Fact Box Only – Expository Text | | Fact Box Only –Narrative Analogy | | Expository Text – Narrative Analogy | |
|  | *H*(2) | *p* | *H*(1) | *p* | *H*(1) | *p* | *H*(1) | *p* |
| **Emotion** |  |  |  |  |  |  |  |  |
| Anger | 0.93 | .629 | 0.39 | .531 | 0.11 | .736 | 0.90 | .344 |
| Fear | 1.35 | .510 | 0.06 | .807 | 0.75 | .387 | 1.21 | .271 |
| **Evaluation of material** |  |  |  |  |  |  |  |  |
| Appealing | 7.89 | .019 | 3.16 | .075 | 7.70 | .006 | 0.92 | .337 |
| Well-made | 6.99 | .030 | 2.46 | .117 | 6.90 | .009 | 1.08 | .300 |
| Reliable | 9.66 | .008 | 8.28 | .004 | 0.07 | .790 | 6.18 | .013 |
| Understandable | 3.29 | .193 | 2.96 | .085 | 0.16 | .693 | 1.83 | .177 |
| Convincing | 3.82 | .148 | 3.44 | .064 | 2.12 | .145 | 0.13 | .716 |
| Interesting | 1.71 | .426 | 1.63 | .202 | 0.29 | .588 | 0.64 | .425 |
| **Attentional focus (fact box)** | 5.08 | .079 | 0.80 | .371 | 5.05 | .025 | 1.76 | .185 |

## Supplement S3

**Table S.4**

*Knowledge Items by Condition (N = 1554)*

| Variable | Condition | | | | | | Group Comparison | | | | | | | | | | | |
| --- | --- | --- | --- | --- | --- | --- | --- | --- | --- | --- | --- | --- | --- | --- | --- | --- | --- | --- |
|  | Fact Box Only | | Expository Text | | Narrative Analogy | | Overall | | | Fact Box Only – Expository Text | | | Fact Box Only – Narrative Analogy | | | Expository Text – Narrative Analogy | | |
|  | *n* | % | *n* | % | *n* | % | Χ*^2^*(2) | *p* | *w* | Χ*^2^*(1) | *p* | *w* | Χ*^2^*(1) | *p* | *w* | Χ*^2^*(1) | *p* | *w* |
| **Item 1: pathogen** |  |  |  |  |  |  | 6.41 | .041 | 0.06 | 5.26 | .022 | 0.07 | 0.06 | .806 | < .01 | 3.86 | < .050 | 0.06 |
| False answer | 170 | 32.02 | 130 | 25.29 | 159 | 31.12 |  |  |  |  |  |  |  |  |  |  |  |  |
| Correct answer | 361 | 67.98 | 382 | 74.61 | 352 | 68.88 |  |  |  |  |  |  |  |  |  |  |  |  |
| **Item 2: vaccination schedule** |  |  |  |  |  |  | 3.35 | .187 | 0.05 | 1.77 | .183 | 0.04 | 2.62 | .106 | 0.05 | 0.04 | .834 | < .01 |
| False answer | 138 | 25.99 | 114 | 22.27 | 110 | 21.53 |  |  |  |  |  |  |  |  |  |  |  |  |
| Correct answer | 393 | 74.01 | 398 | 77.73 | 401 | 78.47 |  |  |  |  |  |  |  |  |  |  |  |  |
| **Item 3: vaccine mechanism** |  |  |  |  |  |  | 25.21 | <.001 | 0.13 | 19.87 | <.001 | 0.14 | 0.16 | .692 | 0.01 | 15.65 | <.001 | 0.13 |
| False answer | 380 | 71.56 | 298 | 58.20 | 359 | 70.52 |  |  |  |  |  |  |  |  |  |  |  |  |
| Correct answer | 151 | 28.44 | 214 | 41.80 | 152 | 29.75 |  |  |  |  |  |  |  |  |  |  |  |  |
| **Item 4: vaccine effectiveness (antigen–virus match)** |  |  |  |  |  |  | 8.45 | .015 | 0.07 | 7.45 | .006 | 0.09 | 4.01 | .045 | 0.06 | 0.43 | .511 | 0.02 |
| False answer | 274 | 51.60 | 220 | 42.97 | 231 | 45.21 |  |  |  |  |  |  |  |  |  |  |  |  |
| Correct answer | 257 | 48.40 | 292 | 57.03 | 280 | 54.79 |  |  |  |  |  |  |  |  |  |  |  |  |
| **Item 5: side effects** |  |  |  |  |  |  | 2.19 | .335 | 0.04 | 1.88 | .170 | 0.04 | 0.86 | .353 | 0.03 | 0.13 | .718 | 0.01 |
| False answer | 100 | 18.83 | 115 | 22.46 | 109 | 21.33 |  |  |  |  |  |  |  |  |  |  |  |  |
| Correct answer | 431 | 81.17 | 397 | 77.54 | 402 | 78.67 |  |  |  |  |  |  |  |  |  |  |  |  |

*Note.* A Χ*^2^*-test was conducted to test for differences in conditions and Cohens *w* stated the effect size.

## Supplement S4

**Table S.5**

*Results of One-factorial ANOVAs for Explorative Dependent Variables*

| Variable | Contrast 1:  Control vs Text | | | | | Contrast 2:  Expository Text vs Narrative Analogy | | | | |
| --- | --- | --- | --- | --- | --- | --- | --- | --- | --- | --- |
|  | Fact Box Only | | Text Plus Fact Box Conditions | | Contrast | Expository Text | | Narrative Analogy | | Contrast |
|  | *M* | *SD* | *M* | *SD* | *F*(1,1551) | *M* | *SD* | *M* | *SD* | *F*(1,1551) |
| Perceived risk of vaccination | 3.26 | 1.66 | 3.19 | 1.68 | 0.65, *p* = .419, $\eta_{p}^{2}$< .01 | 3.18 | 1.69 | 3.19 | 1.66 | 0.01, *p* = .908, $\eta_{p}^{2}$< .01 |
| Intention to get vaccinated | 3.86 | 2.03 | 3.86 | 2.09 | 0.00, *p* = .997, $\eta_{p}^{2}$< .01 | 3.79 | 2.10 | 3.94 | 2.07 | 1.43, *p* = .233, $\eta_{p}^{2}$< .01 |

## Supplement S5

**Table S.6**

*Comparing Correlations Between Conditions*

| Correlated Variables | Correlation Within Condition | | | Comparing Correlations Between Conditions | | | | | |
| --- | --- | --- | --- | --- | --- | --- | --- | --- | --- |
|  | Fact Box Only | Expository Text | Narrative Analogy | Fact Box Only – Expository Text | | Fact Box Only – Narrative Analogy | | Expository Text – Narrative Analogy | |
|  | *r* | *r* | *r* | *z* | *p* | *z* | *p* | *z* | *p* |
| Perceived vaccine effectiveness of preventing Influenza –perceived risk of vaccination | -0.34 | -0.32 | -0.39 | -0.28 | .778 | 1.05 | .295 | 1.32 | .188 |
| Perceived vaccine effectiveness of preventing Influenza –intention to get vaccinated | 0.59 | 0.56 | 0.55 | 0.62 | .535 | 0.95 | .343 | 0.32 | .745 |

## Supplement S6

**Table S.7**

*Results for a Simple Mediation of Perceived Effectiveness between Condition (Control vs Text) and Vaccination Intention*

|  |  |  |  | 95% CI | |  |  |  |
| --- | --- | --- | --- | --- | --- | --- | --- | --- |
| Path | | b | SE(b) | Lower | Upper | B | *z* | *p* |
| a (condition → perceived effectiveness) | | 0.213 | 0.079 | 0.058 | 0.370 | 0.068 | 2.700 | .007 |
| b (perceived effectiveness → intention) | | 0.778 | 0.028 | 0.723 | 0.832 | 0.565 | 28.018 | <.001 |
| Direct c′ (condition → intention) | | -0.166 | 0.090 | -0.342 | 0.010 | -0.038 | -1.856 | .063 |
| Indirect a×b (condition → perceived effectiveness → intention) | | 0.166 | 0.062 | 0.044 | 0.290 | 0.038 | 2.690 | .007 |
| Total (c) | | -0.000 | 0.109 | -0.206 | 0.212 | -0.000 | -0.003 | .997 |

*Note.* Standard errors and 95% confidence intervals of estimates (5000 iterations) are bootstrapped.
